# Excitatory amino acids, possible causative agents of nodding syndrome in eastern Africa

**DOI:** 10.1186/s41182-023-00520-0

**Published:** 2023-05-19

**Authors:** Yasushi Miyauchi, Ayaka Shiraishi, Konami Abe, Yasuaki Sato, Kiyoshi Kita

**Affiliations:** 1 School of Tropical Medicine and Global Health, Nagasaki University, Nagasaki, 852-8523 Japan; 2 Department of Bio Research, Kamakura Techno-Science, Inc., Kamakura, Kanagawa 248-0036 Japan; 3 School of Global Humanities and Social Sciences, Nagasaki University, Nagasaki, 852-8521 Japan; 4 Department of Host-Defense Biochemistry, Institute of Tropical Medicine, Nagasaki University, Nagasaki, 852-8523 Japan

**Keywords:** Nodding syndrome, Kainic acid agonist, Blood brain barrier

## Abstract

**Background:**

Nodding syndrome (NS) is one type of epilepsy and a progressive disease characterized by nodding symptoms with children in sub-Saharan Africa. The burden for NS children is heavy, not only mentally but financially for themselves and their families, and yet, the cause and cure of NS remain unknown. The kainic acid-induced model in experimental animals is a well-known epilepsy model that is useful for studying human diseases. In this study, we examined similarities of clinical symptoms and histological brain changes between NS patients and kainic acid-treated rats. In addition, we argued for kainic acid agonist as one of the causes of NS.

**Methods:**

Clinical signs in rats were studied after kainic acid administration, and histological lesions including the expression of tau protein and gliosis, were examined at 24 h, 8 days, and 28 days after dosing.

**Results:**

Kainic acid-induced epileptic symptoms were observed in rats, including nodding accompanied by drooling and bilateral neuronal cell death in the hippocampus and piriform cortex regions. In the regions that exhibited neuronal cell death, an increase in tau protein expression and gliosis were found immunohistochemically. The symptoms and brain histology were similar in the NS and kainic acid-induced rat models.

**Conclusion:**

The results suggest that kainic acid agonist may be one of the causative substances for NS.

**Supplementary Information:**

The online version contains supplementary material available at 10.1186/s41182-023-00520-0.

## Background

Nodding syndrome (NS) is one form of epilepsy that occurs in children in sub-Saharan Africa. NS appeared in the early 1990s in south Sudan [1–3] and the number of NS patients increased in 2009 in northern Uganda [4, 5], mainly affecting children after 3 years old [3]. Especially in Uganda, the epidemic continued until around 2013, and the number of children who developed NS has reached over 3000 cases [6]. Children who developed NSs were mentally and physically burdened and their families were burdened with regard to care work and household finances, which were major problems. Although in 2017, the number of new cases of NS in northern Uganda has become much lower [7], the persistent incidence of NS has been a major problem because of the long-term care and financial burden on households [8].

The pathogenic mechanism of NS has long been debated, and recently, several researchers have reported that the pathogenic mechanism of NS is due to onchocerca infection [7, 9]. The number of NS patients has decreased with the administration of ivermectin, which is effective for onchocerciasis [7]. In addition, several groups have suggested leiomodin-1-induced autoimmunity [10, 11]. The *Onchocerca* is a parasitic roundworm that has a protein ‘leiomodin-1’, which is homologous to a protein expressed in host neuronal cells that produces an anti-leiomodin-1 antibody in the patient’s blood and attacks neurons [10, 11]. However, there are still questions regarding this onchocerciasis-based hypothesis because of the limited permeability of the antibody in the blood brain barrier (BBB) and the lack of anti-leiomodin-1 antibody in the cerebrospinal fluid of NS patients [12]. Thus it is difficult to consider the cause of NS only in terms of onchocerciasis infection in children, and the involvement of onchocerciasis in NS remains unclear.

There has been an increase in research concerning the brains of children with NS. From the magnetic resonance imaging (MRI) and histopathological analysis of the brains of children with NS, the responsible lesions have been identified in the cerebral cortex, hippocampus and cerebellum and are almost symmetrical [13, 14]. From immunostaining, it was shown that the degenerated neuronal cells in brains with NS are positive for tau protein and exhibit the fibrillar changes commonly associated Alzheimer’s disease [14].

As no clear information of the cause about NS has been available, we focused chemical involvement as one of the causes of NS. In particular, excitatory amino acids, which are possible candidate for the cause of NS, often exist in plants eaten by residents of the epidemic area, and they would be toxic to humans in sufficient quantities. Kainic acid is one of the excitatory amino acids and other excitatory amino acids are glutamic acid, quisqualic acid, ibotenic acid and domoic acid. It is well known that kainic acid causes epilepsy-like symptoms in mice or rats and many characteristics of seizures and behaviors are observed in these kainic acid-induced animal models, for example wet dog shakes, nodding, scratching, and tonic–clonic seizure [15–17]. In addition, kainic acid causes neuronal cell death and gliosis in the cerebral cortex and hippocampus [18], and Purkinje cell degeneration in the cerebellum [19].

Because no reports have shown that excitatory amino acids, including kainic acid, are involved in the pathogenesis of NS, this study examined the similarities between NS and the kainic acid-induced animal epilepsy model in terms of clinical manifestations and histological changes, and investigated the possible cause of NS by kainic acid.

## Materials and methods

### Experimental design

Seven-week-old Sprague-Dawley rats (Charles River Japan, Inc.), weighing 238–258 g, were used in the present study. Each rat was administered intraperitoneally in a single injection with 10 mg/kg (1 mg in 10 ml dissolved in saline control) of kainic acid (Tocris Bioscience) (*n* = 9) and saline (*n* = 3), then clinical signs of each rat were observed. At 24 h (*n* = 3), 7 days (*n* = 3) and 28 days (*n* = 3) after dosing with kainic acid and 28 days (*n* = 3) post-dosing with saline, the animals were euthanized by exsanguination under isoflurane anesthesia. The brain was collected and immediately fixed in a 10% neutral buffered formalin solution for histological analysis and immunohistochemistry.

Animals were housed individually in stainless steel cages under constant conditions of temperature (19–25 °C), humidity (40–60%RH), and lighting (12-h light/dark cycle). Before conducting, the protocol was approved after an ethics review by the Animal Experiment Committee based on the “Rules for Animal Experiments at Kamakura Techno-Science, Inc.” (Approval No.21-003).

### Brain histology

After each brain was fixed in 10% neutral buffered formalin solution, coronal slices of 2 mm thickness were made at each level including the olfactory bulb, cortex, hippocampus, amygdaloid nucleus and cerebellum and embedded in paraffin. Paraffin sections of 3 μm thickness were stained with hematoxylin and eosin (HE) and then examined by light microscopy (Olympus, Japan).

### Immunohistochemistry

Immunohistochemical studies were performed on paraffin-embedded specimens consisting of the cortex, hippocampus, amygdaloid nucleus and cerebellum. From these paraffin-embedded specimens, paraffin sections with a 3 μm thickness were prepared and used for immunostaining with each antigen.

To investigate tauopathy for neuronal injury in rat brains, immunostaining for phosphorylated tau protein was performed using immune-histological methods. In addition, to investigate gliosis in the rat brains, immunostaining for infiltrative astrocytes and microglia was performed using each specific marker; glial fibrillary acidic protein (GFAP) for astrocyte and Iba 1 for microglia. In the case of tau and GFAP, the paraffin sections were deparaffinized in xylene and rehydrated in ethanol, following antigen retrieval by 0.01 M citric acid buffer heated at 121 °C for 5 min using an autoclave and treated with rabbit anti-phosphorylated tau protein (Anti-Tau (pThr231) antibody (sc58-08) (NBP2-67574, Novus Biologicals) or GFAP (M0761, DAKO). In the case of Iba 1, other deparaffinized sections were treated at 90 °C for 9 min using a water heater and with the rabbit antibody against Iba1 (013-27691, FUJIFILM). Antibody binding was detected using MACH 4 HRP-Polymer (Biocare Medical, CA) followed by 3,3′diaminobenzidine staining and then nuclei were stained with hematoxylin. These stained sections were examined by optical microscopy (Olympus, Japan).

## Results

### Clinical symptoms

In rats treated with kainic acid, a decrease in locomotor activity was observed at approximately 20 min after administration, and wet dog shaking was observed at approximately 40 min. In addition, at approximately 90 min to 4 h later, epileptic symptoms of nodding were observed, accompanied by drooling and convulsion (Additional file 1: Video S1), but these changes were not observed from the next day onwards. On the next day after administration, an increase in locomotor activity with each rat was observed, and during the observation period up to 28 days after administration, the rats remained excited. In contrast, rats treated with saline exhibited no symptoms during the breeding period (Additional file 2: Video S2).

### Histopathological changes in the brain

**Fig. 1:**
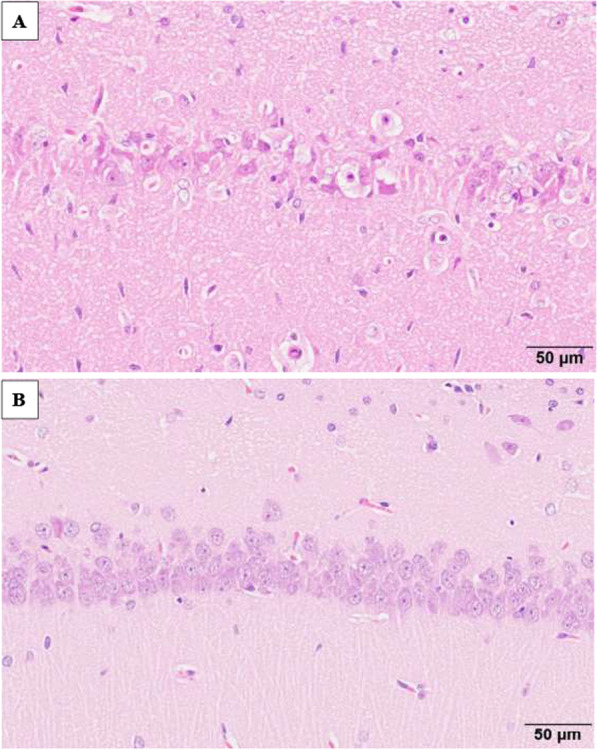
Hippocampus; neuronal cell deaths at Day 28 after administration with kainic acid (**A**) and normal neuron cells with saline (**B**). In **A**, neuronal cell bodies are atrophied and eosinophilic, and the nuclei have disappeared

**Fig. 2:**
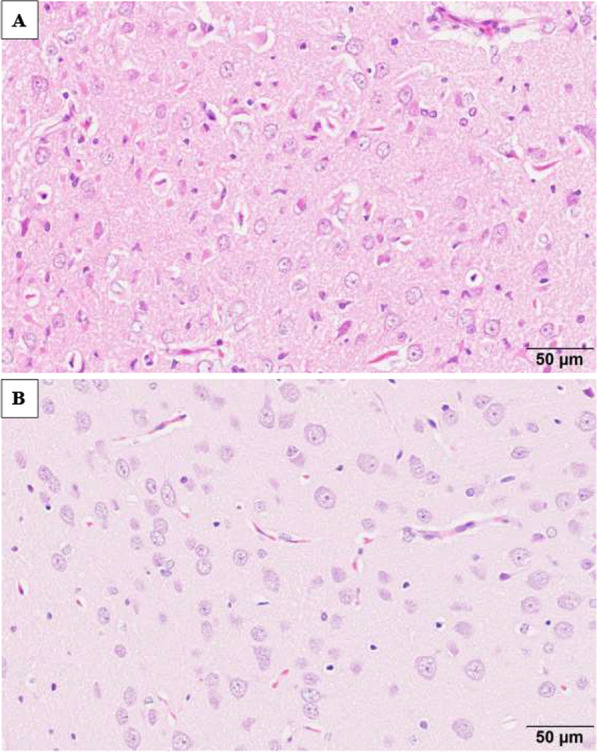
Amygdaloid nucleus; neuronal cell deaths at Day 28 after administration with kainic acid (**A**) and normal neuron with saline (**B**). In **A**, neuronal cell bodies are atrophied and eosinophilic, and the nuclei have disappeared

In rats treated with kainic acid, bilateral neuronal cell death was observed 7 and 28 days after administration primarily in the piriform cortex, hippocampus, amygdaloid nucleus, and thalamic nucleus. Figure 1 shows neuronal cell deaths in hippocampus at Day 28 after administration with kainic acid and Fig. 2 in amygdaloid nucleus.

### Immunohistochemistry

**Fig. 3:**
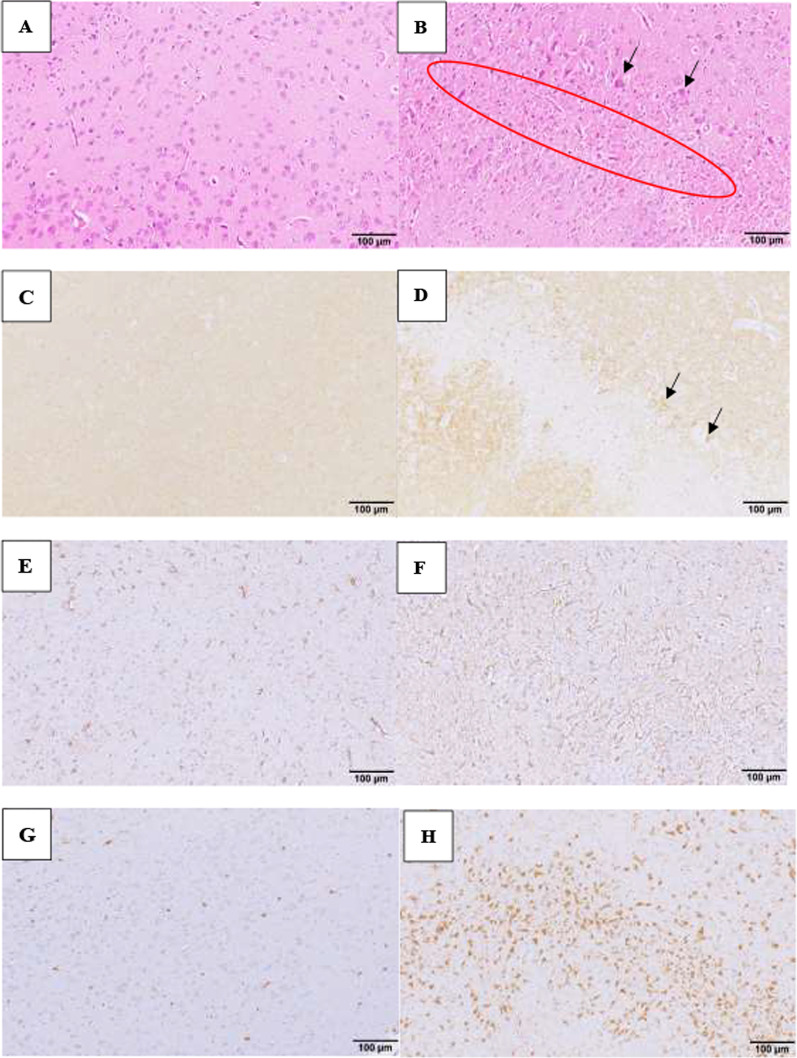
Amygdaloid nucleus; Day 28 after administration with saline (**A**, **C**, **E**, **G**) and kainic acid (**B**, **D**, **E**, **F**), HE staining: (**A**, **B**), expression of phosphorylated tau protein: (**C**, **D**), GFAP: (**E**, **F**), Iba1: (**G**, **H**). **B** Red circle: neuronal cell death, black arrow: neuron around necrosis. **D** Black arrow: increased expression of phosphorylated tau protein in neuron around necrosis. **E** Increase of the number and cell bodies of GFAP-positive cells. **F** Increase of the number and cell bodies of Iba1-positive cells

There was strong expression of phosphorylated tau protein in neurons of each region in rats treated with kainic acid, while a slight expression was found in the saline-treated rats. The increased expression of phosphorylated tau protein was observed in the injured brain areas that exhibited with neuronal cell death (Fig. 3A–D). Even on the day after kainic acid administration, an increase in phosphorylated tau protein was observed, which remained until Day 28. Figure 3D shows an unstained area with an anti-phosphorylated tau protein antibody in the amygdaloid nucleus, and additionally in this unstained area, necrotic neurons were seen. Additionally, the strong expression with phosphorylated tau protein was seen in neurons with relatively well-maintained cell bodies around the unstained area.

To observe the formation of gliosis in response to these neuronal deaths, GFAP-positive astrocytes and Iba-1-positive microglia were examined by immunostaining. Further infiltration of GFAP or Iba-1-positive cells in each region in rats treated with kainic acid was found but there was slight infiltration in the saline-treated rats. GFAP and Iba-1-positive cells were increased in the injured brain areas that had neuronal cell death, especially in the hippocampus and thalamic nucleus on Day 28. As shown in Fig. 3F, the number and size of positive cells increased. In addition, Iba-1-positive cells, like GFAP-positive cells, were increased in number and size, as shown in Fig. 3H.

## Discussion

This study is the first to experimentally demonstrate a possibility that kainic acid is relevant in the pathogenesis of NS. Although there have been numerous studies on the pathogenesis of NS, the cause of disease remains unclear.

In this study, nodding accompanied by drooling, which is one of the typical symptoms observed in children with NS, was observed in kainic acid-treated rats. In children with NS, a repetitive short loss of neck muscle tone appears along with nodding [20]. In addition, such nodding symptoms in NS patients are associated with spikes and slow waves observed by electroencephalography (EEG) [21]. In experiments with animals treated with kainic acid, neck muscle tone and EEG have also been studied in the mechanistic analysis of sleep patterns at that time of epileptic seizures [22–24]. In cats in which kainic acid was administered to the brain and induced neuronal cell death, a loss of neck muscle atonia was observed [22], and spikes and slow waves were observed by EEG in rhesus monkeys treated with kainic acid [23]. These results indicate that neck muscle tone and EEG in the kainic acid-induced animal model have some commonalities with the mechanisms of nodding in children with NS.

Histologically, it is known that kainic acid causes neuronal cell death or degeneration in the cerebral cortex, amygdala, hippocampus and cerebellum in rat brains [18, 19]. In the NS brain, MRI showed that the cerebral cortex, hippocampus, and cerebrum were atrophied, and the gliotic lesions were symmetrically observed in these areas [13]. In the current study, bilateral neuronal cell death accompanied by gliosis was also induced by the administration of kainic acid in similar regions of rat brains. This suggests that kainic acid induces neuronal death in the same brain regions of rats as the brain lesions seen in children with NS. These histological changes in the neuronal cells were still observed in rats 28 days after the administration of kainic acid.

Recently, it was reported that the degenerated neuronal cells in the brains of children with NS are positive for tau protein, the main component of neurofibrillary tangles, and exhibit the fibrillar changes commonly observed for Alzheimer’s disease [14]. Although these aging changes are not usually seen in children’s brains, neuronal cell death may have been progressing over time in children’s brains with NS, similar to Alzheimer’s disease. In this study, while necrotic neurons in the injured regions were unstained with an anti-phosphorylated tau protein antibody, the increased expression of phosphorylated tau protein was observed in the degenerated neuronal cells around the injured regions at 28 days after dosing with kainic acid. These observations are novel and demonstrate that neuronal cell death in this kainic acid-induced rat model progressed slowly over a long period.

In addition to kainic acid, other excitatory amino acids have been reported, including glutamic acid, quisqualic acid, ibotenic acid, and domoic acid. These excitatory amino acids are found in seaweeds, plants, mushrooms, and shellfish. In the case of intake beyond safe limits, toxic effects including neuropathy may be induced in humans. It has been reported that kainic acid is contained in *Digenea simplex*, and this seaweed is used in plant-based drug [25]. Additionally, quisqualic acid is contained in one of the geraniums native to sub-Saharan Africa, and geraniums are usually used in traditional medicine in this region [26, 27]. Quisqaric acid is mentioned as an example and it is reasonable to assume that the local children intake excitatory acids through their diet. For example, l-tricholomic acid contained in *Ustilago maydis* [28], known as maize disease, has an affinity for the kainic acid receptor [29]. Noteworthy is the food history of moldy maize was significantly related to NS in Uganda [30]. It is also possible that the emergency relief food may be contaminated with moldy maize-derived substances since eating emergency food supplies were significantly related to NS in Uganda [30]. Therefore, the ingestion of excitatory amino acids such as kainic acid could be a cause of NS.

In addition, Nakalanga syndrome shows epilepsy-like symptoms similar to NS, and epidemics have been reported in Uganda, Tanzania, Ethiopia, Congo and Cameroon [31, 32], and the population of epilepsy patients is relatively high in Africa [33]. Recently, neuropathological lesions with tau-reactive neurofibrillary tangles were observed in Nakalanga syndrome [34], as well as NS, suggesting slow progressive changes in neuronal cell death in patients with Nakalanga syndrome.

It should be noted that the age at onset of NS is between 3 and 18 years old [3] and onchocerciasis infection has been suggested the main cause of the pathogenic mechanism of NS [7, 8]. Especially in children, the BBB is fragile and highly permeable to drugs and other chemical substances. In this regard, we need to consider the permeability of the BBB in children in NS pathogenesis. Vascular permeability is typically increased during infection, although the relationship between parasite infection and the permeability of BBB has not been studied sufficiently. As one example, *Trypanosoma brucei* infection in sleeping sickness increased vascular permeability [35]. This vascular change may be induced with onchocerciasis infection and as a result, it is conceivable that chemical substances such as kainic acid could easily pass through the BBB to reach the human brain.

In addition, several reports have shown that measles is significantly associated with NS [30, 36] and indicated the importance of the BBB because measles causes progressive encephalitis. Furthermore, association between NS with psychological trauma and stress in children from long-lasting war and conflict has been suggested [36, 37]. The permeability of the BBB increases with mental stress and psychiatric disorders such as autism [38, 39], which may also be involved in the pathogenesis of NS. Thus, it is necessary to investigate the increased permeability of the BBB in the regions of NS onset in terms of its potential action and causes.

In conclusion, the increased permeability of the BBB due to the infectious diseases, including onchocerciasis and measles, and the psychological stress of war and conflict are considered possible causes of NS. The results of this study reveal that nodding symptoms and histological changes in this kainic acid-induced epilepsy model resemble those of NS. The possibility with the excitatory amino acids such as kainic acid and the high permeability of the BBB should be studied further.

**Note added in proof**

After this manuscript was submitted, the following review article was published (Trends in Parasitology, DOI: 10.1016/j.pt.2022.11.010).

## Supplementary Information

**Additional file 1: Video S1**. Nodding symptoms in rats treated with kainic acid.

**Additional file 2: Video S2.** No symptoms in rats treated with saline.

## Data Availability

Not applicable.

## Abbreviations

NS: Nodding syndrome
BBB: Blood brain barrier
MRI: Magnetic resonance imaging
HE: Hematoxylin and eosin
GFAP: Glial fibrillary acidic protein
EEG: Electroencephalography
